# An Anomalous Case

**Published:** 1849-06

**Authors:** William H. Miller

**Affiliations:** Louisville, Ky.


Art. III.—*An Anomalous Case.* By William H. Miller, M.D., of Louisville, Ky.

Susan Curle, aged five years, previously a very healthy child, was attacked, on the 17th of March, with nausea and vomiting, which continued for several days. It then ceased for the time, and an eruption resembling measles appeared, which faded away in three days afterwards. The throat then became swollen, and she had a cough, with pain in the chest, which readily yielded to an expectorant mixture. The child appeared well at this time, with the exception of loss of appetite and occasional vomiting, which continued to recur every few days.

April 11. Visited her for the first time, and found her with considerable fever, cough, and pain in the right side, coated tongue, and constipation of the bowels. No special affection of the chest was discovered by auscultation or percussion; but a very careful examination could not be made on account of her excessive irritability. A mercurial cathartic was prescribed, to be followed by infusion of senna. The next day she had less fever, the purgative medicines having operated well; but she still had cough, and complained of pain, referred rather vaguely to the right side. A simple expectorant mixture, containing squills and antimonial wine, was directed, and the mercurial purgative at night. She was not visited the next day, but on the day following (14th) she still had some fever, and her tongue was coated. The expectorant nauseated more than was intended, and had been discontinued on that account. Her cough had, however entirely ceased; she had frequent inclination to urinate, but voided very little—the urine being of a pinkish color, as if slightly mixed with blood. Spirits of nitre, twenty drops every two hours in watermelon seed tea, was directed, and the bowels to be kept open with castor oil. On the 18th, my attention was directed to a dark spot, which had made its appearance on the outer side of the left leg the day previous, to which a poultice of powdered slippery elm bark had been applied by the mother. A similar spot now appeared on the outer part of the right leg, and spread rapidly towards the knee and ankle, and around the limb. As these spots were the most striking features of the disease, I will endeavor to describe them fully: At their first appearance, they were not larger than a quarter of a dollar, of a dusky red color, and unattended by heat or swelling; they soon became *black*, and vesicated here and there; the surrounding parts also became swollen and tense; the vesications, when punctured or clipped, yielded a bloody serum. Alcoholic tincture of iodine was freely applied to the black spots and the adjacent healthy skin; cloths wet with a liniment of olive oil and lime water were likewise applied; the iodine was directed to be used again at night, and ten grains of Dover's powder, to be repeated, if necessary, to allay pain and procure sleep. The iodine appeared to check the progress of the affection upon the leg; one of these spots then appeared upon the right arm, and spread in the direction of the wrist. It was also apparently controlled by the iodine; but on the 23d black discoloration attacked the pudenda, and spread thence to the nates, and upwards upon the back, as high as the shoulders. Directed a mixture containing one grain quinine every two hours. The progress of the inflammation in this last direction was not at all arrested by the tincture of iodine, although repeatedly and freely used. In the afternoon her countenance was remarkably pale, with a slight greenish tinge, expressive of great suffering, pulse rapid and feeble; she manifested great irritability of temper during the day; the desire to urinate became exceedingly frequent, which was performed with great difficulty and pain; the urine still maintaining its bloody appearance. Directed the free use of wine whey; but she died that night about ten o'clock, having had numerous spasms before the fatal event.

April, 1849.

